# Application of two level count regression modeling on the determinants of fertility among married women in Ethiopia

**DOI:** 10.1186/s12905-022-02060-x

**Published:** 2022-12-09

**Authors:** Nuru Mohammed Hussen

**Affiliations:** Statistics Department, Samara University, Samara, Ethiopia

**Keywords:** Ethiopia, Fertility, Hierarchical data, Multilevel count regression analysis

## Abstract

**Background:**

Fertility is the element of population dynamics that has a vital contribution toward changing population size and structure over time. The global population showed a major increment from time to time due to fertility. This increment was higher in south Asia and sub-Saharan Africa including Ethiopia. So this study targeted the factors affecting fertility among married women in Ethiopia through the framework of multilevel count regression analysis using the 2016 Ethiopian Demographic and Health Survey data.

**Methods:**

Secondary data set on the birth records were obtained from the 2016 Ethiopia Demographic and Health Survey. The survey was a population-based cross-sectional study with a two-stage stratified cluster sampling design, where stratification was achieved by separating every region into urban and rural areas except the Addis Ababa region because it is entirely urban. A two-level negative binomial regression model was fitted to spot out the determinants of fertility among married women in Ethiopia.

**Results:**

Among the random sample of 6141 women in the country, 27,150 births were recorded based on the 2016 Ethiopian Demographic and Health Survey report. The histograms showed that the data has a positively skewed distribution not extremely picked at the beginning. Findings from the study revealed that the contraception method used, residence, educational level of women, women’s age at first birth, and proceeding birth interval were the major predictors of fertility among married women in Ethiopia. Moreover, the estimates from the random effect result revealed that there is more fertility variation between the enumeration areas than within the enumeration areas.

**Conclusion:**

Unobserved enumeration area fertility differences that cannot be addressed by a single-level approach were determined using a two-level negative binomial regression modeling approach. So, the application of standard models by ignoring this variation ought to embrace spurious results, then for such hierarchical data, multilevel modeling is recommended.

## Introduction

Fertility is one of the three most important determinants of a country's population size and structure (the other two being mortality and migration) [1]. The global population showed a five billion increment (2.6 billion to 7.6 billion) from 1950 to 2017 because of this dynamics, wherever most of this increment was higher in South Asia and sub-Saharan Africa relative to the remaining continents of the globe [2].

Ethiopia has a population of nearly 100 million people, making it Africa's second most populous country. The high fertility rate is the basic character of Ethiopian population growth [3]. 44.4% of the total population was under the age of fifteen (CSA, 2016). In step with Ethiopia’s demographic and health survey (EDHS), the country’s fertility rate shows a slight decrement between 2000 and 2005, from 5.9 youngsters per woman to 5.4, so attenuated more to 4.8 youngsters in 2011, then it reaches 4.6 in 2016. The overall wanted fertility rate in Ethiopia is 3.0 youngsters per woman, 1.6 less than the total fertility rate in 2016 [4]. Even though fertility has shown a declining trend at the national level, the transition has not begun in several regions. There were clear regional variations in fertility levels and trends in Ethiopia. Like other developing countries, vital variation in fertility level was ascertained among rural and urban residents of Ethiopia. For example in keeping with the 2016 Ethiopian demographic and health survey report, on average, rural women can offer birth to just about 3 more kids throughout their generative years than urban women(5.2 versus 2.3 kids per woman) [5].

The Ethiopian government has been creating many efforts to cut back fertility levels since 1993. The primary time a certain national population policy geared toward reducing the total fertility rate from 7.7 youngsters per woman to 4.0 by 2015 was launched [6]. Increasing the age at first marriage to a minimum of eighteen years, enhancing women’s standing by providing them with higher employment and academic opportunities, and increasing birth control services and knowledge, communication, and education on the ways and suggest that limiting family size were some of the ways designed to implement the population program [7]. Fertility trends will be foreseen and an increase will be enrolled by recognizing the factors that affect fertility preferences and desires. Within the setting of the above circumstances, this study attempted to spot out the factors related to the total number of children ever born among married women in Ethiopia. Many studies investigated determinants of fertility in Ethiopia with some sets of variable and statistical methods like logistic regression, survival analysis, and linear regression models [8–11]. Since the total number of children ever born is a count data, the Poisson regression model (PRM) and Negative binomial regression models (NBRM) are shown to be statistically more acceptable. However, on the far side of the count nature of the data, it has hierarchical nature wherever women are nested beneath the enumeration area and every enumeration area were nested beneath the region. All the above standard statistical models and techniques have problems in handling the multilevel data structure, for example in regression models violating the independence and normality assumption of errors with a constant variance [12–14]. Heteroscedasticity raises if the underlying dependency that comes attributable to the multilevel nature of the data isn’t adjusted within the simple regression models. In such cases, multilevel models rather than standard models got to introduce for considering the direct impact of the individual and also group-level variables. There are several studies that restricted multilevel models for the analysis of hierarchical data [15, 16]. Hence with respect to those pieces of literature, this study geared towards factors affecting fertility among married women in Ethiopia through the framework of multilevel analysis using the Ethiopian demographic and health survey (EDHS) data.

## Methods

### Data source and study design

The present paper uses secondary data set from the 2016 Ethiopia Demographic and Health Survey (EDHS) which was the fourth demographic and health survey. From January 18, 2016, to June 27, 2016, the survey used a population-based cross-sectional study design in which women were interviewed for information on their birth history questionnaires. EDHS was strengthened by the Central Statistical Agency (CSA) with support obtained from the Ministry of Health. This survey was carried out in Ethiopia, under the worldwide measure of the DHS project, a USAID-funded project that provides support and technical assistance with the implementation of population and health surveys in countries around the world.

### Sampling design

The sampling frame for EDHS 2016 was the same as for EDHS 2007, which was previously conducted by the Central Statistical Agency in 2007 (CSA, 2008). This survey uses a census frame of 84,915 enumeration areas (EAs) created for the 2007 PHC. An EA could be defined as a geographic area covering a mean of 181 households. Administratively, Ethiopia is divided into 9 nation-states and 2 administrative cities. Every region is sub-divided into zones, every zone into Woredas, every woreda into cities, and every city into kebeles. For the EDHS 2016, a two-stage stratified cluster sampling design was used. Every region was stratified by dividing it into urban and rural areas. In total, 21 sampling strata were created as a result of the Addis Ababa region being entirely urban. Samples of EAs were selected independently in every stratum in 2 stages. Among the primary stage 645 selected EAs, 202 were urban and 443 were from rural areas in the second stage of selection, a fixed number of 28 households per cluster were chosen through an equal probability systematic selection from the newly created household listing. Each one among women aged 15–49 and each one among men aged 15–59, who were either permanent residents of the chosen households or visitors who stayed within the household the night prior to the survey, were able to be interviewed.

### Variables in the study

The response variable of this study was the total number of children ever born per women in selected enumeration areas. It is a count variable from the birth record dataset in EDHS 2016. However, the contraception method used, Women’s educational level, Wealth index, Preceding birth interval, Religion, and Women’s age at first birth were potential lower level, predictors of fertility, while residence was a potentially higher level predictor of fertility. Secondary data was managed with SPSS-26 and analyzed using R-3.6.3. Even though the data on the number of children per women is suitable for analysis, weighting of the data was applied to increase representative ness of the sample and correcting for non-responses. In this study, multiple imputations was used as missing data handling mechanisms. The model selection was made using LRT for nested, AIC, and BIC for non-nested models.

### Statistical analysis

Count regression models analyze the data with integer outcome variables. These models may be used to look at the prevalence or frequency of prevalence over time. Various models were developed for the analysis of count data [17, 18]. These models can cope with the dependent variable's non-normality and don't require the researcher to transform or dichotomize the variable. The four most popular models in this study were the Poisson regression model, the Negative Binomial model, the Zero-inflated Poisson model, and the Zero-inflated Negative Binomial model [19, 20].

### Poisson regression model

The most common model for count data is a Poisson regression model, which is based on the assumption that the dependent variable's mean and variance are equal [21]. Let ${, Y}_{i}$ represent counts of events occurring in a given time or exposure periods with rate ${\mu }_{i} {, Y}_{i}$ are Poisson random variables with probability mass function;

$$P\left(Y_i\mathit=\mu_i\right)=\frac{e^{-\mu_i}\mu^{y_i}i}{y_i!};\;\mu_i>0,\;i=1,\;2,\;\dots,\;n\;y_i=0,\;1,\;2,$$

Where ${y}_{i}$ denotes the total number of children ever born for the ${i}^{th}$ women in a given time or exposure period with parameter ${\mu }_{i}$

In the Poisson model, the conditional variance is equal to the conditional mean:

$$E\left({Y}_{i}\right)=Var\left({Y}_{i}\right)={\mu }_{i}$$

This property of the Poisson distribution is known as equi-dispersion.

Let X be $n X (P+1)$ covariate matrix**.** The relationship between ${Y}_{i}$ and ${i}^{th}$ row vector of X,

${x}_{i}$ is given by the Poisson log-linear model; $\mathrm{ln}\left({\mu }_{i}\right)= {{x}_{i}}^{T}\beta = {\eta }_{i}$

Where ${x}_{i}=\{{1,{x}_{i1}, {x}_{i2}, \dots , {x}_{ip }\}}^{T}$ is the vector of explanatory variables and $\beta =({{\beta }_{0}, {\beta }_{1}, \dots , {{\beta }_{p}}\}}^{T}$ is the vector of unknown regression parameters.

### Negative binomial regression model

Most of the time, the count variable with over dispersion is modeled by the negative binomial regression model which is more flexible than the Poisson model [21–23]. The probability mass function for a negative binomial random variable is given by;

$$p\left({y}_{i},{\upmu }_{i},\alpha \right)=\frac{\Gamma ({y}_{i}+\frac{1}{\alpha })}{{y}_{i!}\Gamma (\frac{1}{\alpha })}{(1+\alpha {\upmu }_{i})}^{-\frac{1}{\alpha }}{(1+\frac{1}{\alpha {\upmu }_{i}})}^{{-y}_{i}}, {y}_{i>0}$$

where $\alpha$ is the over-dispersion parameter and $\Gamma (.)$ is the gamma function when$\alpha =0$. The negative binomial distribution adds an explicit error term ε to the Poisson regression model, as follows; ${\mu }_{i }=$ exp(${\eta }_{i}+ {\upvarepsilon }_{i}$) = exp(${\eta }_{i}$)* exp(${\upvarepsilon }_{i}$), where ${\eta }_{i}=$ ${{x}_{i}}^{T}\beta$

Then the negative binomial log-linear model is given by; $\mathrm{ln}\left({\mu }_{i}\right)= {{x}_{i}}^{T}\beta +{\upvarepsilon }_{i}$

Where ${x}_{i}=\{{1,{x}_{i1}, {x}_{i2}, \dots , {x}_{ip }\}}^{T}$ the vector of explanatory variables, $\beta =({{\beta }_{0}, {\beta }_{1}, \dots , {{\beta }_{p}}\}}^{T}$ is the vector of unknown regression parameters, and ${\upvarepsilon }_{i} \mathrm{is an}$ explicit error term (dispersion parameter) added to the Poisson regression model.

### Two-level count regression model

The multilevel Poisson regression model for a count ${Y}_{ij}$ on the ${i}^{th}$ level 1 in ${j}^{th}$ level 2 with C varying exposure rate can be written as: ${Y}_{ij}/{\lambda }_{ij}$ = Poisson(${C}_{ij}$,${\lambda }_{ij}$). Then the two-level Poisson regression model containing p lower level and q higher level predictors is given by;

$${\text{In}}\left({\lambda }_{ij}\right)={\upeta }_{ij}={\beta }_{00}+{\sum }_{i=1}^{p}{\beta }_{i0}{x}_{ij}+{\sum }_{j=1}^{q}{\beta }_{0j}{Z}_{j}+{\sum }_{j=1}^{q}{\sum }_{i=1}^{p}{\beta }_{ij}{x}_{ij}{Z}_{j}+{U}_{0j}+{\sum }_{j=1}^{q}{\sum }_{i=1}^{p}{U}_{ij}{x}_{ij}$$

In the Poisson regression model, the variance of the outcome variable is assumed to be equal to the mean. When the observed variance is much larger than expected under the Poisson model, we have over-dispersion. One way to model over-dispersion is to add an explicit error term to the model. Then the negative binomial model adds an explicit error term ε to the model, as follows;

$$\left(\mu_{ij}\right)\;=\;exp\left(\eta_{ij}+\varepsilon_{ij}\right)=\;exp\left(\eta_{ij}\right)\ast exp\left(\varepsilon_{ij}\right)$$

Then the two-level negative binomial log-linear regression model is given by;

$$\mathrm{ln}\left({\mu }_{ij}\right)= {\eta }_{ij}+{\upvarepsilon }_{ij}$$

$$={\beta }_{00}+{\sum }_{i=1}^{p}{\beta }_{i0}{x}_{ij}+{\sum }_{j=1}^{q}{\beta }_{0j}{Z}_{j}+{\sum }_{j=1}^{q}{\sum }_{i=1}^{p}{\beta }_{ij}{x}_{ij}{Z}_{j}+{U}_{0j}+{\sum }_{j=1}^{q}{\sum }_{i=1}^{p}{U}_{ij}{x}_{ij}+{\varepsilon }_{ij}$$

Where; γ_00_ represents the overall log rate of birth across all women and enumeration areas.

u_0j_ represents the residual errors at the enumeration area with respective variances as ${\upsigma }^{2}{u}_{0}$

$\beta_{i0}\;and\;\beta_{0j}$ Represent lower level and higher level fixed effects respectively with corresponding predictors.

${\beta }_{ij}$ represents the interaction effect of higher level and lower level predictors on the response variable and ${\upvarepsilon }_{ij}$ represent the error term that increases the variance compared to the Poisson regression model.

## Results

**Fig. 1:**
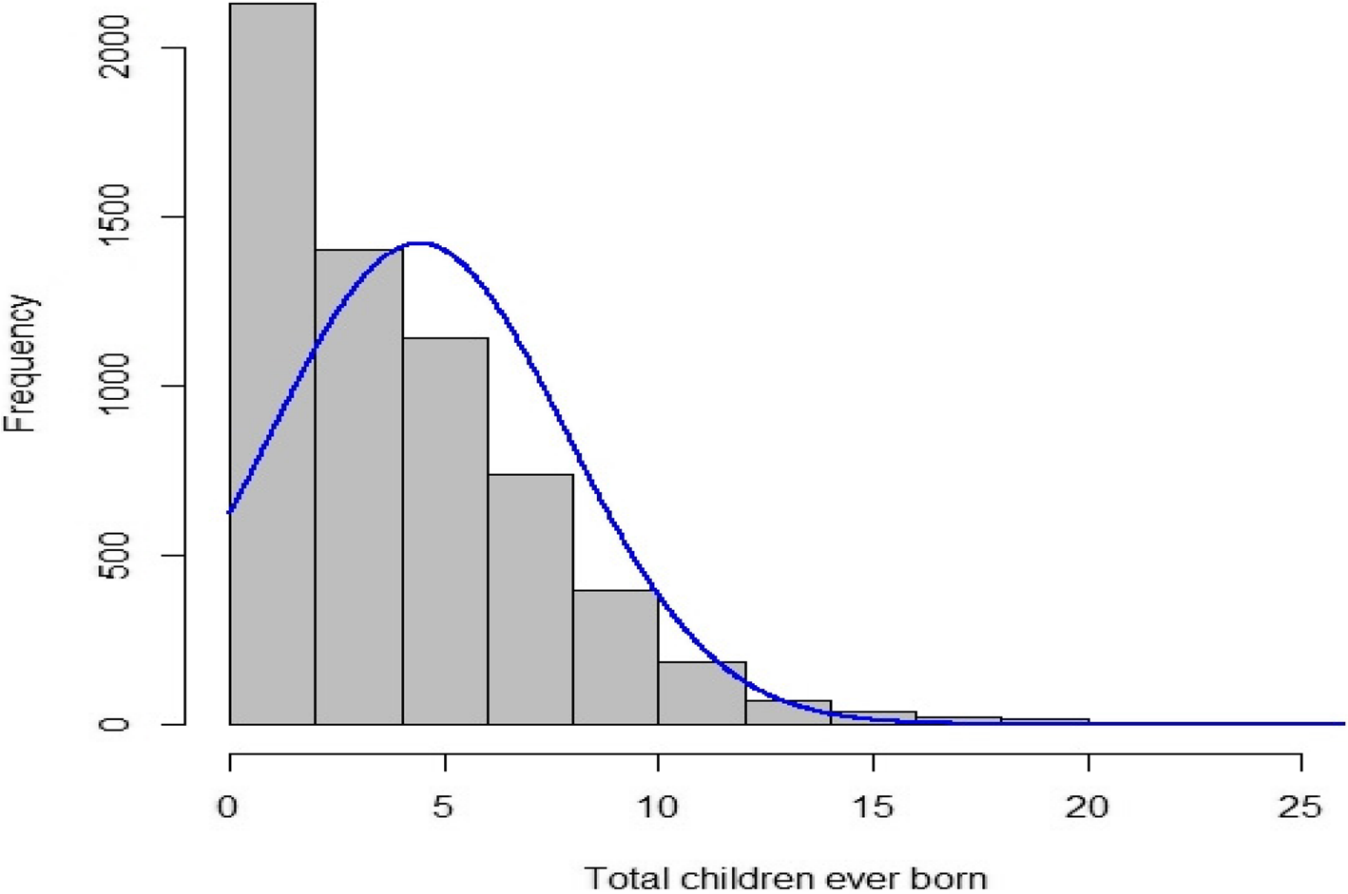
Histogram for the numbers of children ever born

According to the EDHS 2016 report, 27,150 births were reported among the randomly selected 6141 women in the country. The histograms were not extremely picked at the beginning, but higher observations (larger number of children per woman) were observed less frequently. This ended up in a positively skewed distribution of the data. Extra screening of fertility showed that the variance (11.873) was higher than the mean (4.42), which implies the existence of over-dispersion, this means the data could be fitted better by a negative binomial regression model (Fig. 1).

### Number of children ever born and its socio-demographic and economic features

The majority, 77.3% of women were selected from rural areas of Ethiopia. Most of the women (52.2%) used traditional methods of contraception, while 44% of them did not use any method, while the remaining (3.8%) uses modern methods of contraception. The majority of these sample women were illiterate (58.3%), Muslim (40.9%), and poorest income level (35. 4%) relative to other corresponding labels of a factor (Table 1).

**Table 1:** Descriptive Statistics

Variable	Categories	Frequency	Percent
Place of residence	Urban	1393	22.7
	Rural	4748	77.3
Age at first birth	In year		
Contraception method	Not used	2705	44
	Modern	233	3.8
	Traditional	3203	52.2
Women’s educational level	Illiterate	3578	58.3
	Primary	1774	28.9
	Secondary	481	7.8
	Higher	308	5
Religion	Orthodox	2386	38.8
	Catholic	39	0.6
	Protestant	1140	18.6
	Muslim	2508	40.9
	Traditional	39	0.6
	Others	29	0.5
Wealth index	poorest	1575	25.6
	Poorer	1040	16.9
	middle	955	15.6
	Richer	953	15.5
	Richest	1618	26.4
Proceeding birth interval	month		

### Model selection criteria

Since the histogram for the number of children ever born is not extremely at the beginning the data are not zero-inflated, so we cannot apply zero-inflated models. The negative binomial regression model has a minimum value for the fit statistics than the Poisson regression model. Consequently, it is selected as the best model for the total number of children ever born among married women in Ethiopia (Table 2).

**Table 2:** Model Selection

Fit Statistics	Models	
	Poisson	Negative Binomial
**AIC**	22,553.6349	22,080.5602
**BIC**	22,669.6574	22,203.0284

### Fitting two-level negative binomial regression model

When we are interested to analyze on a single level by standard regression models the model fitting is quite easy, but when we have hierarchical data with the predictors at different levels, the process of model fitting should consider a series of steps. While the most crucial step for multilevel analysis is fitting the model with no predictors (i.e. the intercept-only model).

$${\mathrm Y}_{\mathrm{ij}}={\mathrm\gamma}_{00+}{\mathrm u}_{0\mathrm j+}{\mathrm e}_{\mathrm{ij}}\;\mathrm{where}:$$

Y_ij_ represents the total number of children ever born from ${i}^{th}$ women in ${j}^{th}$ enumeration area,

γ_00_ represents the overall log rate of birth across all women and enumeration areas.

u_0j &_ e_ij_ represent the residual errors at the enumeration areas and women level respectively with their respective variances as ${\upsigma }^{2}{u}_{0} and {\upsigma }^{2}{\varepsilon }_{0}$ where $\varepsilon_{ij} \sim N(0,\sigma_{\varepsilon }^{2} )$

The residual error variance at the women and enumeration area level was estimated to be 0.2937& 0.5748 respectively as a result of the maximum likelihood estimation. We can conclude that the overall rate of birth across all women and all enumeration areas was 4.42 and that there is more variation among the different enumeration areas (0.5748) than within the enumeration areas (0.2937). This will be discussed further when we calculate the intra-class correlation (*ρ*) for this model below. Intra-class correlation represents the proportion of variance explained due to the grouping structure.

$$\rho =\frac{{\sigma }^{2}{u}_{0}}{{\sigma }^{2}{\varepsilon }_{0}+{\sigma }^{2} {u}_{0}}=\frac{0.5748}{0.2937+0.5748}=0.66$$

This result implied that 66% of the fertility variation was at the enumeration area level and the remaining 34% was among women (Table 3).

**Table 3:** Intercept only model

Estimates for fixed effects							
Effect	$\hat{\beta}\;\text{Exp}\;(\hat{\beta}))$	SE	t Value	Pr >|t|	Alpha	Lower	Upper
Intercept	1.4864(4.42)	0.009945	149.47	< .0001	0.05	1.4669	1.5059
Estimates for random effects							
Covariance Parameter				Estimate			
Intercept(U_oj_)				0.5748			
Scale(e_ij_)				0.2937			

The final two-level random intercept and slope negative binomial regression model was fitted through all the significant predictors in the univariable analysis. Accordingly, among the predictors in the final model contraception method used, residence, educational level of women, women’s age at first birth, and the proceeding birth interval were the major predictors for fertility among married women in Ethiopia. Moreover, the estimates from the random effect result revealed that there was more variation between the different enumeration areas (0.06512) than within the enumeration areas (0.05255), furthermore, the variation among rural and urban areas was more significant (0.08982) in the number of children ever born among married women in Ethiopia (Table 4).

**Table 4:** Final two-level negative binomial regression model

Solutions for Fixed Effects							
Variable	Categories	$\hat{\beta}$	SE	$\text{Exp}(\hat{\beta}))$	Pr >**|**t**|**	Lower	Upper
Intercept		1.4474	0.1063	4.25	< .0001	1.2389	1.6558
Contraception use	No	0.1747	0.0393	1.19	< .0001	0.09764	0.2517
	modern method	0.1500	0.0185	1.16	< .0001	0.1138	0.1862
	traditional method	0					
Residence	Rural	0.1322	0.0344	1.14	0.0001	0.06482	0.1996
	Urban	0					
Wealth index	Poorest	0.005339	0.0343	1.00	0.8763	-0.06190	0.07258
	Poorer	0.01027	0.0353	1.01	0.7713	-0.05900	0.07955
	Middle	0.02772	0.0355	1.03	0.4346	-0.04182	0.09726
	Richer	0.07319	0.0347	1.08	0.0352	0.005083	0.1413
	Richest	0					
Religion	Catholic	0.03312	0.1351	1.03	0.8064	-0.2318	0.2980
	Muslin	0.09213	0.0872	1.10	0.2905	-0.07872	0.2630
	Orthodox	0.02384	0.0881	1.02	0.7867	-0.1489	0.1965
	Other	0.07837	0.1381	1.08	0.5704	-0.1924	0.3491
	Protestant	0.08597	0.0887	1.09	0.3325	-0.08792	0.2599
	Traditional	0					
Education level	No education	0.4157	0.0460	1.52	< .0001	0.3256	0.5058
	Primary	0.1788	0.0467	1.20	0.0001	0.08734	0.2703
	Higher	-0.0514	0.0712	0.95	0.4705	-0.1911	0.08824
	Secondary	0					
Age at first birth		-0.0016	0.0004	0.10	< .0001	-0.00248	-0.00102
Proceeding birth interval		-0.0121	0.0016	0.99	< .0001	-0.01521	-0.00897
**Solutions for random effects**							
**Covariance Parameter**				**Estimate**			
Intercept **(U**_**oj**_**)**				0.06512			
Residence				0.08982			
Scale **(e**_**ij**_**)**				0.05255			

## Discussion

In this study, secondary data from 6141 randomly selected women were assessed using a two-level negative binomial regression model, and significant factors associated with fertility among married women in Ethiopia were identified.

The estimated fixed intercept was 1.4474, which represents the overall rate of birth was *e*^1.44474^ = 4.25 (*p*-value < 0.0001) across all the enumeration areas and all women. Based on the result of this study, contraception use has a significant effect on the rate of birth among married women in Ethiopia, accordingly, when all other factors were held constant, the birth rate among women who did not use contraception and those who used modern means of contraception was 1.19 and 1.16 times higher, respectively, than the birth rate among those who used traditional methods of contraception. The result is coherent with the study done by [24, 25] where ever use of contraception reduced the rate of birth. The rate of birth among women from rural areas was 1.14 times the rate of birth among women from urban areas, the result was consistent with the study conducted by [8, 9, 25–27] where rural women desire to have more children than urban women. The rate of birth among women with no and primary education was 1.52 and 1.2 respectively times the rate of birth with secondary education, moreover, no statistically significant difference was observed in the rate of birth by women with higher and secondary education levels. The result is conformable with [11, 25, 26, 28] where increasing the educational level of women reduced the rate of birth. A one-year increase in the age at first birth of women results in a 0.0017 decrement in the log rate of birth, the result is consistent with the finding of [11, 25, 26, 29]) where the older the age at first birth of women the lower number of children ever born per woman will be. Additionally, a one-month extension of a woman's preceding birth interval caused a 0.01209 reduction in the log rate of birth, which was consistent with the findings of [30, 31], where increasing proceeding birth interval resulted in the reduced number of children ever born.

## Conclusions

Unobserved enumeration area fertility differences that cannot be addressed by a single-level approach were determined using a two-level approach. Additional screening of the data showed that the variance of an outcome was greater than the mean which is an implication of over-dispersion, then the data could be fitted better by a negative binomial regression model. Inferentially, the negative binomial regression model fits the data well due to the minimum value for the fit statistics than the remaining models. The intercept-only model demonstrated that there is more variation among the different enumeration areas than within the enumeration areas. Contraception method used, residence, women's education level, women's age at first birth, and preceding birth interval were significant predictors of fertility among married women in Ethiopia at a 5% level of significance in the final two-level negative binomial regression model. The use of standard models by ignoring higher-level variation could embrace spurious results, so multilevel medaling is recommended for such hierarchical data.

### Limitations

This study was a two-level analysis of factors associated with fertility in Ethiopia, this is due to the inaccessibility of regional-level factors. So, policymakers of CSA should include regional-level variables for better future results.

## Data Availability

The data set used for this study was women’s data from EDHS 2016. The data was accessed from the Measure DHS website (http://www.measuredhs.com).

## Abbreviations

AIC: Akaike Information Criteria
BIC: Bayesian Information Criteria
CSA: Central Statistical Agency
EA: Enumeration Area
EDHS: Ethiopian Demographic and Health Survey
GLM: General Linear Regression Model
ICF: International Consultancy Fund
LL: Log-Likelihood
LRT: Likelihood Ratio Tests
NB: Negative Binomial
NBRM: Negative Binomial Regression Models
PRM: Poisson Regression Models
SE: Standard Error
TWFR: Total Fertility Rate
USAID: United States Agency for International Development

## Declarations

The author affirms that this study was a result of his own real work. All documents used for writing it were appositely acknowledged.
